# A scoping review of the nurse practitioner workforce in oncology

**DOI:** 10.1002/cam4.769

**Published:** 2016-06-05

**Authors:** Lorinda A. Coombs, Lauren Hunt, Janine Cataldo

**Affiliations:** ^1^ University of California San FranciscoSan FranciscoCalifornia

**Keywords:** Cancer, nurse practitioner, oncology, scoping review, workforce

## Abstract

The quality of cancer care may be compromised in the near future because of work force issues. Several factors will impact the oncology health provider work force: an aging population, an increase in the number of cancer survivors, and expansion of health care coverage for the previously uninsured. Between October 2014 and March 2015, an electronic literature search of English language articles was conducted using PubMed^®^, the Cumulative Index to Nursing and Allied Health Sciences (CINAHL^®^), Web of Science, Journal Storage (JSTOR^®^), Google Scholar, and SCOPUS^®^. Using the scoping review criteria, the research question was identified “How much care in oncology is provided by nurse practitioners (NPs)?” Key search terms were kept broad and included: “NP” AND “oncology” AND “workforce”. The literature was searched between 2005 and 2015, using the inclusion and exclusion criteria, 29 studies were identified, further review resulted in 10 relevant studies that met all criteria. Results demonstrated that NPs are utilized in both inpatient and outpatient settings, across all malignancy types and in a variety of roles. Academic institutions were strongly represented in all relevant studies, a finding that may reflect the Accreditation Council for Graduate Medical Education (ACGME) duty work hour limitations. There was no pattern associated with state scope of practice and NP representation in this scoping review. Many of the studies reviewed relied on subjective information, or represented a very small number of NPs. There is an obvious need for an objective analysis of the amount of care provided by oncology NPs.

## Introduction

The quality of cancer care may be compromised in the near future because of work force issues. Several factors are poised to significantly impact the oncology health provider work force: an aging population, an increase in the number of cancer survivors, and expansion of health care coverage for the previously uninsured.

The number of Americans 65 years or older will grow to an unprecedented number, more than doubling between 2010 and 2050 1. As a large proportion of the United States grows older, cancer incidence and prevalence rates are expected to rapidly increase 1. Cancer is the second leading cause of death in the United States 2, and disproportionately affects adults aged 65 years and older 3. Although there are modifiable risk factors that contribute to developing cancer, (e.g., smoking, viral exposure, and physical activity) 4, one of the greatest risk factors is aging. The increased risk of developing cancer with age is linked to deoxyribonucleic acid (DNA) methylation changes that impact gene silencing and activation with age;^5^ unlike other cancer risk factors, DNA methylation changes are not greatly affected by behavioral change. Moreover, earlier detection and improved cancer treatments have extended life expectancy, the number of cancer survivors are increasing. Currently, there are 14.5 million cancer survivors and by 2024, that number will increase to 19 million 6. In addition, as a result of the Affordable Care Act (ACA), millions of previously uninsured Americans now have insurance and access to health care increasing the demand for services 7.

In 2014, the American Society of Clinical Oncologists (ASCO) released a report titled “The State of Cancer Care in the United States,” noting that not only are the number of cancer diagnoses expected to increase, but access to cancer care is unequal and anticipated oncologist shortages could have a further negative impact on care 6. ASCO anticipates that there will be a shortage of 1500 oncologists and the shortage could be exacerbated by other factors including early physician retirement due to higher levels of burnout 6. The 2014 report revised an earlier 2010 workforce analysis that had projected a higher shortage of oncologists 8. Using an input–output model of oncology and radiation oncology services, ASCO estimates a 40% growth in demand by 2025, but only a 25% growth in physician supply in the same time period 7. Physician shortages in primary care have been addressed by utilizing nurse practitioners (NP) to fill the workforce gap 9, a similar model may succeed in oncology.

## Advanced Practice Provider Workforce

The NP workforce has grown significantly since the first registered nurse (RN) completed advanced training in 1965 10. The Health Resources and Service Administration (HRSA) conducted a National Sample Survey of NPs (NSSNP) in 2012, and reported a total of 154,000 licensed NPs 11. The American Academy of Nurse Practitioners (AANP) currently reports the total number of nurse practitioners (NPs) in the United States. at 205,000 12.

Advanced practice providers were included in the 2015 ASCO report; these providers were defined as NPs, Doctors of Nursing Practice (DNP), and Physician Assistants (PA) 6. The results of the practice survey reported that 2700 DNP/NPs were employed 6, no further specific information on advanced practice providers was available. The authors noted in the report that NPs and DNPs were able to prescribe chemotherapy and, at the time of publication, had independent practice in 20 states. Since the report was published, the number of states where NPs have independent practice has increased from 20 to 22 states 13.

The National Center for Health Workforce Analysis predicted a significant growth of advanced practice nurses (APN) between 2010 and 2025, with physician growth estimates at 21% and APN at 141%. Included in the survey of APNs were as follows: certified registered nurse anesthetists (CRNA), NPs, and certified nurse midwives (CNM) 14 (Table 1). While many of the NPs surveyed provided primary care, a large number worked in surgical and internal medicine specialties, such as oncology. The APN growth is anticipated to be particularly significant in the nonprimary care areas (i.e., specialties and subspecialties). The 2012 HRSA NSSNP reported that 31% of the current NP workforce provides specialty care (Table 2). The total number of NPs providing care in oncology was not separated from other internal medicine specialties.

**Table 1:** Nonprimary care and subspecialty clinician workforce composition

Workforce role year	2010(%)	2025(%)
Physicians	73	59
Advanced practice nurses	19	30
Physician assistants	8	11

Adapted from: 2014 Health Resources and Service Administration Non‐Primary Care Specialty and Subspecialty Clinical Supply Projections to 2025.

**Table 2:**
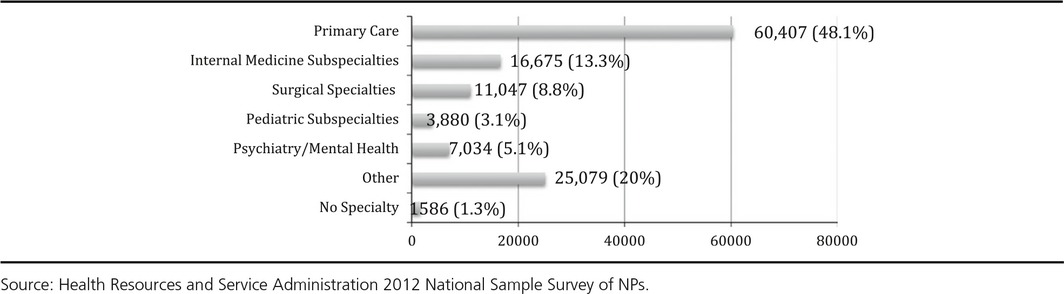
Specialty of practice/facility for NPs providing patient care

Despite the evidence of increasing numbers of NPs providing oncology care, there has not been a systematic review of the literature to evaluate the quantity of care NPs deliver to adults in oncology. Therefore, the purposes of this review are to: describe the amount of the oncology care provided by NPs to adults with cancer, describe the amount of care given to older adults with cancer by NPs, and characterize the economic impact of the care delivered by NPs.

## Scoping Review Methodology

Although there is clear evidence that NPs are providing care in oncology 15, 16, 17, a thorough review of the literature describing that care has not been conducted. Initially, a systematic review of the literature was planned. The systematic review methodology was selected to minimize bias, reduce chance effects, and provide a clear and transparent process. However, after refining the initial results (confirmed with a second, reviewer), the research study designs included observational, quasi‐experimental, and randomized controlled trials. The dissimilarity of the study designs and lack of overlapping outcome variables prohibited completion of a systematic review. Because the research topic has not been extensively studied previously, the decision was made to conduct a scoping review of the literature.

### Scoping review

A scoping review is a method of reviewing the literature that synthesizes knowledge, incorporates multiple study designs, and summarizes the findings with the goal of informing practice, impacting policy, and identifying future research priorities 18. Scoping reviews summarize research findings when the topic has not been extensively studied 19. In contrast to systematic reviews that focus on randomized controlled trials, scoping reviews may include a diverse range of study designs and methodologies 18.

In 2005, the first framework for scoping reviews was proposed by Arksey and O'Malley and involved five steps: identify the research question, identify the relevant studies, select studies, chart data, and summarize results. The optional sixth step was consultation with stakeholders that may involve perspectives different from the data included 18. According to Daud (2013), “Scoping studies aim to map the literature on a particular topic or research area and provide an opportunity to identify key concepts, gaps in the research, and types and sources of evidence to inform practice, policymaking, and research”. The current scoping review framework includes: identification of the research question in a broad manner, identification of relevant studies in as comprehensive process as possible, selection of studies with an established inclusion/exclusion criteria, extraction of data, and a descriptive results summary 20.

## Methods

### Identify the research question

Between October 2014 and March 2015 an electronic literature search of English language articles was conducted using PubMed^®^ (National Center for Biotechnology Information, U.S. National Library of Medicine, Bethesda, MD), the Cumulative Index to Nursing and Allied Health Sciences (CINAHL^®^; EBSCO Information Services, Ipswich, MA), Web of Science, Journal Storage (JSTOR^®^; JSTOR, New York, NY), Google Scholar, and SCOPUS^®^ (Elsevier Inc. (Corporate Office) Marquis One, Atlanta, GA). Using the scoping review criteria, the research question was identified “How much care in oncology is provided by NPs?” Key search terms were kept broad and included: “NP” AND “oncology” AND “workforce”.

Following the scoping review framework, multiple databases were used to produce a comprehensive list of relevant studies. The search resulted in 2120 studies in Google Scholar, 168 studies in JSTOR ^®^, 20 studies in PubMed^®^, 9 studies in Web of Science, 2 studies in SCOPUS^®^, and 0 studies from CINAHL^®^. A total of 2319 studies were evaluated by title and year. Gray literature was included in the search and resulted in an additional four studies for a cumulative total of 2323 studies.

Gray literature has been defined as “non‐conventional, fugitive, and sometimes ephemeral publications. They may include: reports, theses, conference proceedings, bibliographies, technical and commercial documentation, and official documents not published commercially” 21.

### Identification of relevant studies

Since the focus of the scoping review was to assess the quantity of care provided by NPs to patients with cancer in the United States., the search was limited to studies done in the United States. The diagnosis of cancer was a required inclusion criteria, and studies were included if the patient population sampled had comorbidities in addition to a cancer diagnosis, for example, congestive heart failure (CHF), chronic obstructive pulmonary disease (COPD), human immunodeficiency virus (HIV), and end stage renal disease (ESRD).

The focus of the scoping review was NPs in oncology; other advanced practice registered nurses such as clinical nurse specialists (CNS), CRNAs, and certified nurse midwives (CNM) were not included in the review. Although CNMs, CRNAs, and CNSs may be involved in providing care to patients with a cancer diagnosis, their role is typically limited to procedures (CRNA), pregnancies (CNM), or patient education (CNS), and are not a usual source of care for oncology patients. If studies included PAs with NPs in their analysis, they were included in the review; however, studies that focused only on PAs only were excluded.

Because the first significant oncology workforce report that included NPs was published in 2005 22, the literature was searched from 2005 through 2015. Given the temporal and adaptive nature of the oncology workforce supply and changing demands due to an aging population, gray literature, and additional studies that were identified by searching bibliographies, abstracts, and poster presentations were included.

After eliminating duplicates and application of inclusion and exclusion criteria to the abstracts, 29 studies remained. To minimize study selection bias in the literature search, a second blinded reviewer was given a 10% sample of the 2323 research articles with the inclusion and exclusion criteria and asked to perform a review of the titles and abstracts. Using the inclusion and exclusion criteria, the reviewer's search of the abstracts yielded the same 29 studies; confirming a lack of bias in the search strategy.

Further review of the 29 full texts resulted in a total of 10 studies that met all of the inclusion and exclusion criteria. Data extracted from these studies included: (1) outcome variables measured, (2) study design, (3) data used and method of data collection, (4) provider type (e.g., NP, PA) and total number, (5) patient population, (6) malignancy type, (7) setting (e.g., ambulatory, academic, private, inpatient), and (8) state scope of practice.

Initially, age group and economic impact were included in the assessment tables. However, specific information on age groups was consistently not available and it was not included in the analysis. Only one study included a productivity analysis of NP and PA care, with no associated financial data. Because no information was available to analyze the economic impact of NP involvement in patient care, it was also not included in this review.

Four tables divided by study design were developed and includes: six cross‐sectional studies (Table 3), two randomized controlled trials (Table 4), one quasi‐experimental study (Table 5), and one retrospective cohort (Table 6).

**Table 3:** Results summary—cross‐sectional studies

Study	Dependent variable focus	Design and data used method of data collection	Provider type specialty or subspecialty *n* = total providers	Malignancy type	Setting (ambulatory or inpatient) private or academic	State (scope of practice)
Britell 2010	Identify NP and PA function in WA, e.g., type of practice, work role, research participation	Cross‐sectional self‐report survey, 50% response rate	NPs and PAs—Response to survey may not have been identified providers	Not specified	25 total = 8 single specialty, 7 multi‐, 6 hospital based, and 4 academic	WA—full practice
Friese 2010	Practice and physician characteristics that employed NP/PAs	Cross sectional over 2 years (6/2005 to 2/2007) using SEER data mailed survey to physicians in L.A. and Detroit	Not specified	Breast cancer	Both private and academic, setting not distinguished	MI and CA—restricted practice
Hinkel 2010	Identify how NCI‐designated cancer centers use NP/PAs and pilot a productivity tool.	Cross sectional, convenience sample from NCI Cancer Centers. Online survey 4/2004‐5/2004 for 4‐hour clinic block. Only 176 were included in productivity analysis (Med Onc, Heme/BMT, SurgOnc).	206 NPs/PAs NP = 111, PA = 95, Med Onc = 71 (34%), Heme/BMT = 57, SurgOnc = 48, RadOnc = 6 NeuroOnc = 4, Palliative = 4, Others = 4	All included	All academic affiliated NCI Cancer Centers, both inpatient and outpatient.	15 NCI centers in 13 states.Restricted practice: CA, FL, MA, MI, TX, Reduced practice: AL, MO, NY, PA, UTFull practice: NE, WA
McCorkle 2012	Formulate recommendations for enhancing NP/PA roles within multidisciplinary teams	Cross‐sectional study online survey of NPs and PAs in NCI Cancer Center from 10/12/2010 to 11/4/2010 included MD surveys, focus groups, and “consultation with outside experts”	32 NP/PAs sampled (19 NPs and 13 PAs) in NCI‐designated Cancer CenterBreast = 11 Heme = 11 Lung = 10 G.I. = 9 Head/Neck = 8 CNS = 7 Melanoma = 6 GU = 4 GYN=4 Sarcoma = 3	All included.	Both—at one Northeastern NCI Cancer Center rebuilding hospital.	CT—full practice
Moote 2011	Collect data on NP/PA use in academic medical centers	Cross‐sectional study of UHC affiliated academic centers (107) and hospitals (233)—Response rate of 35%—Survey conducted from 7/2009 to 9/2009 included organizational assessment by COO, CMO, chief PA/NP	Of the 26 centers responding, 24 (92% of sample) reported using NPs in oncology.14 (54%) reported using PAs in Onc	Not mentioned in study	Both 26 ACGME centers from across the country with varied states (map of responding centers in article).	Varied, depending upon region of ACGME reporting.
Vichare 2013	Assess radiation oncology workforce	Cross‐sectional data from 2012 ASTRO online workforce survey19% response rate = 6765 out of 35,000	1047 Radiation oncologists, 1231 radiation therapists, 890 dosimetrists, 1105 physicists, 93 NPs, 25 PAs, 484 RNs	Any malignancy requiring radiation.	21% academic, 25.2% hospital, 53.3% private	All states

**Table 4:** Results summary—randomized controlled trial studies

Study	Dependent variable focus	Design and data used method of data collection	Provider type specialty or subspecialty *N* = total providers	Malignancy type	Setting (ambulatory or inpatient) Private or Academic	State (scope of practice)
Bakitas 2009	Resource use with NP palliative care telephone intervention, secondary outcome‐patient mood.	RCT of 322 newly diagnosed stage IV cancer patients (stage III for lung cancer) Enrolled 11/03–5/07	NPs—two with palliative care training	All types	Ambulatory care in academic institution	NH—full practice
Dyar 2012	QOL and hospice knowledge change from baseline (using FACT‐T) with NP palliative care intervention.	RCT of planned 50 patients (enrollment closed after Bakitas study results) accrued 26 patients. Used FACT‐G and LASA instruments for baseline and 1 month after NP intervention	NPs—one with palliative care training	Noted in analysis breast = 12, lung = 2, prostate = 1, others = 11	Ambulatory care in academic institution	FL—restricted practice

**Table 5:** Results summary—quasi‐experimental

Study	Dependent variable focus	Design and data used method of data collection	Provider type specialty or subspecialty *N* = total providers	Malignancy Type	Setting (ambulatory or inpatient) Private or academic	State (scope of practice)
Buswell 2009	Assess provider and patient satisfaction with three different visit models (shared visit model SVM, independent visit model IVM, mixed visit model MVM). measure productivity and revenue	11 teams of NP/PA/MDs followed up for 3 months and 1‐year retrospective fee analysis for revenueThe specific numbers of NPs/PAs and MDs not detailed. Patient satisfaction from 68 patient interviews. Fees and patient visits (new and established) revenue generation was measured by technical fees, only professional fees for MDs	11 teams that included six NPs	Not specified.	Ambulatory academic setting	MA—restricted practice

**Table 6:** Results summary—retrospective study

Study	Dependent variable focus	Design and data used method of data collection	Provider type specialty or subspecialty *N* = total providers	Malignancy type	Setting (ambulatory or inpatient) private or academic	State (scope of practice)
Chandak 2014	Evaluate oncology workforce changes in Nebraska over 5 years	Retrospective analysis of health professions tracking data maintained by University of Nebraska (relies on self‐report and semiannual hospital/clinic surveys)	Medical, surgical, and radiation oncology.37 NPs126 MDs25 PAs	Not specified.	Both private and academic, both ambulatory and inpatient	NE—full practice

## Results

### Description of the studies

The outcome variables in the 10 included studies of this scoping review are diverse. They range from: (1) provider and patient satisfaction assessment, (2) NP function, (3) recommendations for enhancing NP roles, (4) identification of practice and physician characteristics that employ NPs, and (5) assessment of NPs in palliative care interventions. The diverse range of variables examined demonstrates the need for a comprehensive assessment of the oncology care currently provided by NPs.

As shown in Table 3, six of the 10 studies in this review were cross sectional and their focus was on identification and collection of data on NP function, recommendations for NP role enhancement, and assessment of the NP presence in radiation oncology. The number of NPs included in the sample was difficult to identify, since three of the studies did not include specific information on the number of providers, and instead reported the percentage of centers that utilized NPs 23, 24, 25. The sample of NPs included in the scoping review ranged from one to 111, and of the seven studies that reported the number of NPs, six had 37 NPs or fewer in their sample.

As shown in Table 4, two of the 10 studies in this review were randomized controlled studies and focused on NPs provision of palliative care. One study measured patient resource utilization with a telephone intervention, and the other used a patient quality of life measurement and hospice knowledge changes from baseline to establish the impact of a NP palliative care intervention. Although the total number of NP providers in the study was not included in the report, an e‐mail inquiry to the study authors confirmed the number of NPs included in both studies was three.

As shown in Tables 5 and 6, the remaining two studies were quasi‐experimental (Table 5) and retrospective (Table 6) in design. The quasi‐experimental study assessed provider and patient satisfaction with three different visit models and included six NP providers in the study. The longitudinal study evaluated oncology workforce changes in Nebraska over 5 years and included 37 individual NP providers.

## Discussion

The important findings of this scoping review include: (1) An accurate estimation of NP care in oncology does not currently exist; (2) Many of the studies included in this review had methodological problems due to a reliance upon self‐report and small sample sizes; (3) The total number of NP providers included in this review were 269 out of 154,000 licensed NPs 11, only 0.1% of the licensed NP population in the United States. were represented; (4) Academic settings were more likely to utilize NPs than private practice settings; (5) NP providers were present equally among inpatient and outpatient settings, there was also no evidence that certain oncology specialties (e.g., breast, lung, bone marrow transplants, etc.) had disproportionate NP representation; and finally (6) There was no concordance between state scope of practice and the number of NPs in the oncology workforce.

### No accurate estimation of the number of NPs in oncology care

The main aim of the review was to quantify the care provided by NPs to patients with cancer. Several studies evaluated the aspects of NP care, that is, recommendations for role enhancement 26, NP presence in radiation oncology 27, NP function 25 and practice characteristics that employ NPs; 24 but no studies evaluated the amount of care provided. The lack of a comprehensive study surveying the full scope of NP care in oncology severely limits the ability to answer the primary research question. Without accurate data on the NP oncology workforce, it is impossible to address to what degree or even whether their contribution could impact the anticipated oncology physician deficit.

### Problematic methodology

Four of the 10 studies relied completely upon either written or online self‐report surveys 25, 26, 27, 28 without independent verification of the information. Of the remaining six studies, two relied upon proxies (administrators, practice manager, or physician) to report data on NPs; 23, 24 only four studies had objective data on NP numbers and practice 29, 30. The four studies that included verified data on NP care in oncology account for 46 of the total number of 269 NPs included in all of the studies in this scoping review. The small number of NPs (i.e., three) represented in the randomized controlled studies, illustrate the uneven quality of studies conducted on NP oncology care in the United States. Four of the largest studies in this scoping review represented 89 percent of the NPs and relied upon a single online survey to gather data 25, 26, 27, 28.

Several of the studies had only one or two NP providers in the sample.

(Table 4) 31, 32 and the complete scoping review results are based on 269 NPs (93 from the ASTRO radiation oncology workforce survey), representing only 0.1% of the NPs in the United States. The small number of NPs represented in the research coupled with the significant projected increase in specialty NP care by the Center for Health Workforce Analysis illustrates the gap in knowledge of NP practice in oncology.

### Setting

Academic institutions were included in all of the 10 studies reviewed. The strong representation of NPs in the academic oncology workforce may be a result of the impact from the resident duty work hour limitation imposed by the Accreditation Council for Graduate Medical Education (ACGME). In 2003, the ACGME set the resident work hour limit at an 80‐hour week, this new standard reduced the number of hours residents were available to provide patient care. In addition to the reduced work hours, the ACGME also mandated one day off a week from patient care, further reducing the workforce labor provided by residents 33. Although private practices were included in several of the studies 24, 27, 29, the lack of a specific analysis on how much care was provided by NPs in the private practice environment prohibits further generalizations. Nine out of the 10 studies included both inpatient and outpatient settings; this suggests that the impact of ACGME reduced work hours has impacted both outpatient ambulatory oncology patient care as well those patients requiring hospitalization.

### Malignancy subtypes and inpatient/ambulatory care settings

There was evidence of NP care of patients across all malignancy subtypes, and no evidence of a dominant trend within any specific solid tumor or hematologic malignancy type. Only one study 24 specifically focused on the employment of NPs and PAs in providing breast cancer care. This may be a result of the data not being included in the analysis, or, more likely that NPs are utilized throughout multiple different oncology specialties. There was also equal representation of inpatient and outpatient care settings, among the studies included in the scoping review.

### State scope of practice

**Figure 1:**
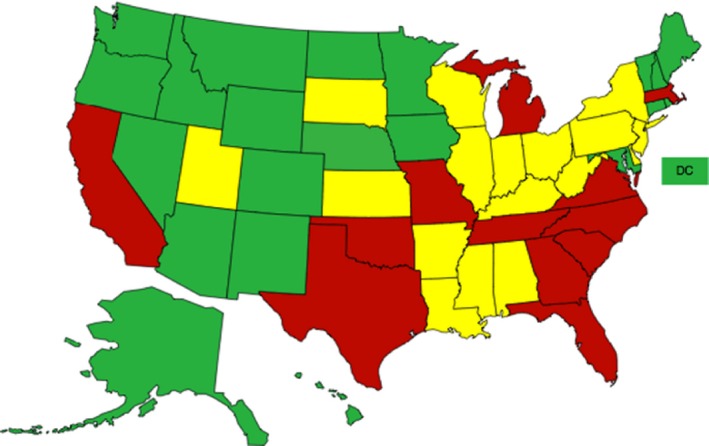
AANP 2015 NP State Practice Environment: 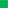 full practice, 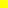 reduced practice, 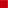 and restricted practice Source: State Nurse Practice Acts and Administrative Rules 2015 Updated 5.20.2015.

Scope of practice was included in the analysis to assess if any pattern of NP patient care emerged across the scope of practice spectrum. Scope of practice was defined according to the AANP simplified definition, separating NP practice into three categories: full, reduced, and restricted (Fig. 1) 13.

Full practice was defined as “state practice and licensure law provides for NPs to evaluate patients, diagnose, order, and interpret diagnostic tests, initiate and manage treatment—including prescribing medications—under the exclusive licensure authority of the state board of nursing” 13. Reduced practice was defined as “state and licensure law reduces the ability of NPs to engage in at least one element of NP practice. State requires a registered collaborative agreement with an outside health discipline in order for the NP to provide patient care.”^13^ And restricted was defined as “state practice and licensure law restricts the ability of a NP to engage in one element of NP practice. State requires supervision, delegation, or team management by an outside health discipline in order for the NP to provide patient care” 13.

There was no pattern of increased NP use in any of the three categories of practice: four states were represented in full or independent practice (CT, NE, NH, WA), five studies were completed in reduced practice states (AL, MO, NY, PA, UT), and five states were represented in restricted practice (MI, FL, MA, TX, CT). Although some primary care literature has suggested that full scope of practice encourages NP practice, this finding was not supported in evaluating NP care in oncology.

## Scoping Review Strengths and Limitations

A strength of this review was the use of a second blinded reviewer for 10% of the total abstracts and titles from the initial search strategy. While the addition of a third reviewer to evaluate complete articles may have enhanced the methodological rigor of this review, the benefit may have been limited. It is possible that the exclusion of results prior to 2005 may have reduced the overall number of relevant research studies included in this review. The lack of a formal quality assessment instrument prohibits a specific measurement, but the overall rating for the design, methodology, and analysis of the included studies is fair.

## Conclusion and Recommendations

This scoping review offers an examination of current knowledge on the oncology NP workforce. Significant gaps in the literature exist: on the number of NPs providing oncology care, the amount of care provided, and on the amount of care delivered to older adults. There is also great variation in the NP provider role, evident from the wide range of NP functions in the studies included in this scoping review.

Recommendations for future research include an accurate, comprehensive identification of the NP workforce, an objective analysis of the amount of care provided, and an evaluation of the financial impact of NP care in oncology. Given the established presence of NPs in oncology, the predicted growth of older adults who will require increased amounts of care and the anticipated deficit of oncologists; an accurate portrait of the NP workforce in oncology is critical.

## Conflict of Interest

None declared.
